# Endothelial barrier disorder in hereditary angioedema: molecular mechanisms and therapeutic implications

**DOI:** 10.3389/fimmu.2026.1853949

**Published:** 2026-06-01

**Authors:** Nan Zhou, Jianqiang Wu, Yuxiang Zhi

**Affiliations:** 1 Department of Allergy and Clinical Immunology, Peking Union Medical College Hospital, Chinese Academy of Medical Sciences and Peking Union Medical College, Beijing, China; 2 Institute of Clinical Medicine, National Infrastructure for Translational Medicine, Peking Union Medical College Hospital, Chinese Academy of Medical Sciences and Peking Union Medical College, Beijing, China; 3 State Key Laboratory of Complex Severe and Rare Disease, Peking Union Medical College Hospital, Beijing, China; 4 National Clinical Research Center for Immunologic Diseases, Beijing Key Laboratory of Precision Medicine for Diagnosis and Treatment on Allergic Diseases, Beijing, China

**Keywords:** biomarkers, Bradykinin, endothelial barrier, Endothelial permeability, glycocalyx, hereditary angioedema

## Abstract

Hereditary angioedema (HAE) is a rare genetic disorder characterized by recurrent episodes of vascular leakage and tissue swelling. Although excessive bradykinin generation has long been considered the central pathogenic mechanism, increasing evidence indicates that endothelial cells play a decisive role in determining when and where vascular permeability occurs. This Review summarizes recent advances in endothelial biology relevant to HAE, highlighting how intercellular junctions, the endothelial glycocalyx, and dynamic endothelial activation states cooperatively regulate barrier integrity. Newly identified HAE subtypes caused by pathogenic variants that directly affect endothelial regulatory pathways further support endothelial dysfunction as a key disease mechanism beyond bradykinin excess. By integrating bradykinin-dependent and bradykinin-independent processes within an endothelial-centered framework, this Review proposes a revised conceptual model for HAE pathogenesis and discusses its implications for biomarker discovery and therapeutic strategies aimed at stabilizing the endothelial barrier.

## 1 Introduction

Hereditary angioedema (HAE) is a rare autosomal dominant disease marked by recurrent, unpredictable edema of the skin, airway, or gastrointestinal tract, with laryngeal attacks posing a risk of fatal asphyxiation (1). HAE affects an estimated 1:50,000–100,000 individuals worldwide, and the true prevalence is likely higher given its rarity and clinical heterogeneity (2–4). Misdiagnosis is common, leading to global diagnostic delays of approximately 10 years, which extend to approximately 12.6 years in China (5–7).

Recent research has indicated that endothelial barrier dysregulation in HAE is not merely a passive consequence of excess bradykinin (BK) but also an active determinant of edema formation. Excess BK and related mediators remodel endothelial junctions, disrupt the glycocalyx, and induce cytoskeletal reorganization, resulting in transient paracellular leakage. In addition, several endothelial-derived or vasoactive molecules exhibit characteristic alterations during both attack and remission, suggesting they are potential biomarkers of endothelial activation and disease activity (8–10). The identification of additional HAE-associated gene variants that affect endothelial signaling further suggests that endothelial dysfunction may represent a primary pathogenic mechanism in specific subtypes (11, 12). Consistent with this evolving concept, the DANCE classification recognizes vascular endothelium dysfunction-related angioedema as a distinct category, alongside mast cell-mediated, BK-mediated, drug-induced, and unknown-etiology angioedema (13).

Although previous reviews have extensively addressed the genetic basis, plasma contact system/kallikrein–kinin system (CAS/KKS) activation, BK generation, and current therapies in HAE, an integrated endothelial-centered framework remains lacking. In particular, the endothelial compartment is often treated as a downstream target of BK rather than as an active regulator of edema susceptibility. This review therefore summarizes endothelial barrier regulation, BK–endothelial signaling, glycocalyx disturbance, and endothelium-associated HAE subtypes, with a focus on their implications for vascular permeability and therapeutic intervention.

## 2 Complexity of the endothelium

Endothelial cells (ECs), which constitute a single-cell layer across all organs, are often perceived as a rather inert cell population. However, the vascular endothelium should be considered a highly dynamic and interactive organ with systemic dissemination. Rather than merely serving as a passive physical barrier between the bloodstream and surrounding tissues, the endothelium functions as an active regulator, continuously modulating key cardiovascular processes (14).

The endothelium integrates diverse signals from blood components, hormones, neurotransmitters, and surrounding cells, coordinating adaptive responses to environmental changes and ensuring vascular homeostasis. This ability to interpret and respond to complex signaling cues highlights the central role of the endothelium in maintaining vascular permeability, regulating blood flow, and modulating immune responses (15).

### 2.1 Composition and molecular regulatory mechanisms of the endothelial barrier

Endothelial cells regulate vascular permeability to facilitate substance exchange between the circulatory system and surrounding tissues. Fluid movement from the intravascular space to the interstitial space mainly involves diffusion and filtration, whereas the transport of proteins and macromolecules is controlled via interendothelial junctions (paracellular pathway) or through biochemical transporters, fenestrae, and vesicular systems (transcellular pathway) (16, 17).

Cell junction systems play a central role in maintaining barrier integrity. Tight junctions are composed primarily of claudins, occludins, and junctional adhesion molecules, which regulate the static sealing properties of ECs [14]. In contrast, adherens junctions offer greater plasticity for dynamic regulation, with vascular endothelial cadherin (VE-cadherin) as a key component (18, 19). VE-cadherin forms a “pericellular zipper-like structure” along the cell boundary through its cis and trans interactions (20) and binds to catenins (including p120-catenin, β-catenin, and α-catenin) via its C-terminal cytoplasmic tail, anchoring the complex to the actin cytoskeleton and linking it to several signaling pathways (21, 22). This complex not only maintains the stability of VE-cadherin at the cell membrane, preventing proteolytic degradation, but also facilitates the dynamic opening and closing of adherens junctions through the regulation of kinases (such as Src and Yes) and phosphatases (23–27). Under the influence of inflammatory factors (such as cytokines and chemokines), VE-cadherin can be rapidly internalized, leading to the transient opening of intercellular gaps and increased vascular permeability (19).

### 2.2 Regulation of vascular function by endothelial permeability

Changes in endothelial permeability directly affect tissue perfusion and blood pressure regulation. Short-term blood pressure is controlled by neuroendocrine mechanisms, whereas long-term regulation depends on systemic fluid balance (28). ECs secrete a variety of vasoactive molecules to regulate peripheral vascular tone. Among them, nitric oxide (NO) synthesis is among the most important vasodilatory mechanisms and is mediated by endothelial nitric oxide synthase (eNOS) (29). NO induces vasodilation by activating smooth muscle guanylate cyclase, reducing intracellular calcium levels. Additionally, endothelial-derived vasodilatory molecules include prostaglandin E2, prostacyclin, and the putative endothelium-derived hyperpolarizing factor (30). Vasoconstrictor factors, such as endothelin, can cause abnormal vasoconstriction under pathological conditions, where endothelin receptor antagonists are typically employed (31).

Additionally, ECs secrete anticoagulant factors, modulate platelet activation, and regulate fibrinolysis to prevent thrombosis. They also mediate vascular angiogenesis in response to vascular endothelial growth factor (VEGF), ensuring adequate tissue perfusion (32, 33). Moreover, ECs regulate the expressions of adhesion molecules, control leukocyte infiltration, and participate in immune and inflammatory responses to maintain tissue immune defense (34).

Disruption of endothelial permeability regulation can lead to a range of pathological consequences, including edema, increased interstitial pressure, vascular leakage, and impaired drug delivery (35). Traditionally, “endothelial dysfunction” refers primarily to the loss of endothelial regulation of vascular resistance, often accompanied by chronic structural damage, as observed in conditions such as pulmonary hypertension and neurodegenerative diseases (36). However, in certain diseases, endothelial morphological and functional changes are transient, with nearly complete restoration following the resolution of the acute phase. For example, endothelial dysfunction in sepsis is driven primarily by functional alterations—such as increased leukocyte adhesion, vasomotor dysregulation, loss of barrier function, and hemostatic imbalance—although reversible structural changes, including cytoplasmic swelling and endothelial cell detachment, may also occur (37).

In HAE, excessive BK generation induces transient endothelial activation during attacks, causing reversible barrier disruption and vascular leakage; this attack-related process is distinct from chronic endothelial dysfunction characterized by persistent structural or functional impairment. On this basis, the concept of paroxysmal permeability disorder (PPD) was proposed. This disease category is characterized by recurrent alterations in endothelial permeability without inflammatory, degenerative, or ischemic vascular injury and with complete recovery after each episode. PPDs encompass primary angioedema types (including HAE, acquired angioedema, idiopathic histaminergic and nonhistaminergic angioedema); idiopathic systemic capillary leak syndrome; and other less-defined forms of periodic edema (38).

## 3 Overview and pathological mechanisms of HAE

**Figure 1:**
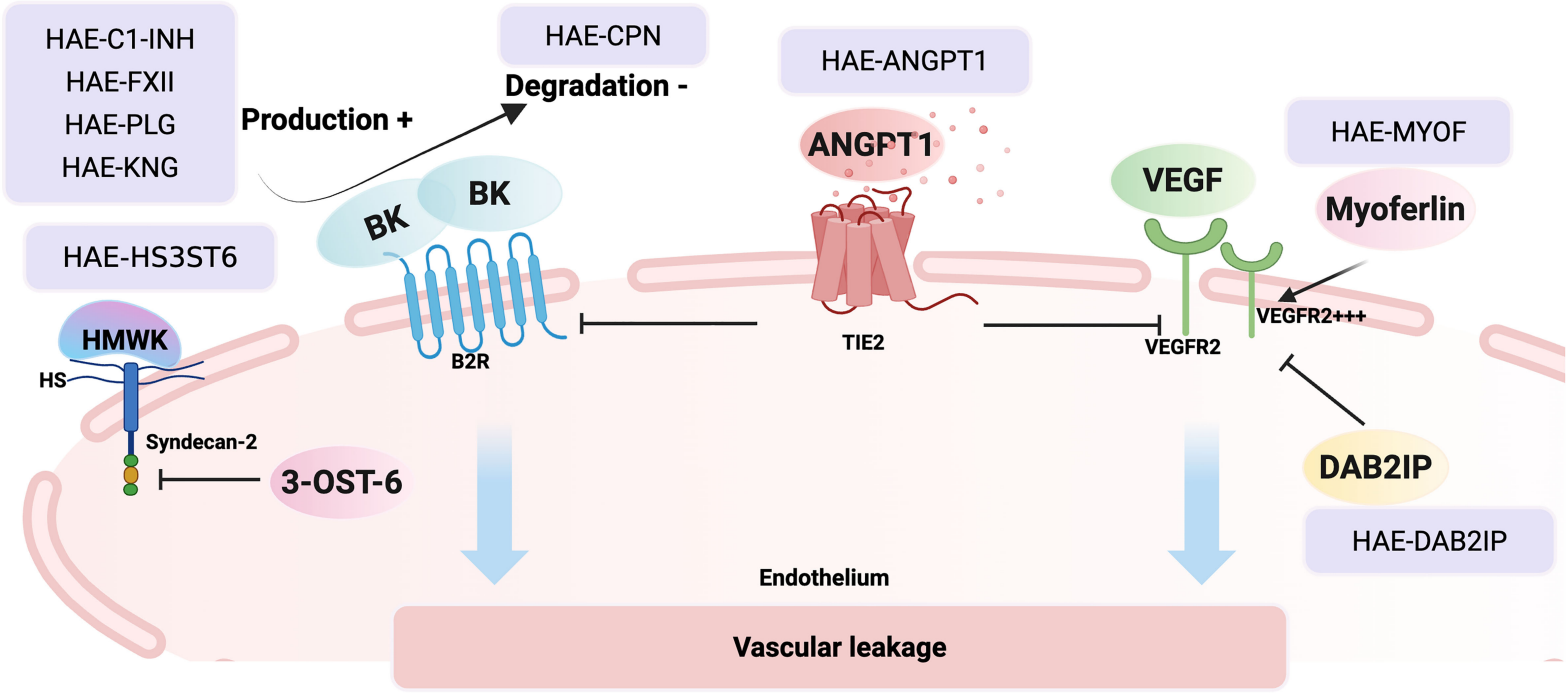
Crosstalk among the contact system, complement system, and coagulation/fibrinolytic pathways in the pathogenesis of HAE. The upper panel summarizes the interactions among the contact system, complement system, and coagulation/fibrinolytic pathways in hereditary angioedema (HAE). The principal inhibitory targets of C1 inhibitor (C1-INH) are indicated in red. Deficiency of functional C1-INH leads to dysregulated activation of factor XII (FXII), plasma kallikrein (PKa), and complement-associated serine proteases, resulting in sustained contact system activation. High molecular weight kininogen (HMWK) is cleaved by PK/PKa to release bradykinin (BK), which acts on endothelial B2 receptors to induce vasodilation and increased vascular permeability. The lower panel illustrates the zinc ion (Zn²^+^)-dependent assembly of the plasma contact system on the endothelial surface. FXII, HMWK, and prekallikrein (PK) are anchored via a multireceptor complex consisting of gC1qR, cytokeratin 1 (CK1), and uPAR, facilitating FXII activation and subsequent bradykinin release.

HAE can be categorized into two types: C1 inhibitor deficiency (HAE-C1-INH) and non-C1 inhibitor deficiency (HAE-nC1-INH). HAE-C1-INH is attributed to *SERPING1* mutations, which encode the C1 inhibitor (C1-INH) (3). As a key serine protease inhibitor, C1-INH deficiency permits varying degrees of uncontrolled activation of its target enzymes. In the complement pathway, C1-INH deficiency allows autoactivation of C1r and C1s, triggering the complement cascade and excessive cleavage of C4 and C2 (39, 40). In the contact system, unopposed factor XII (FXII) activation generates FXIIa, which converts prekallikrein (PK) to activated plasma kallikrein (PKa), leading to extensive cleavage of high-molecular-weight kininogen (HMWK) into BK (41, 42). Excess BK binds to endothelial receptors and induces the production of vasoactive mediators, promoting smooth muscle relaxation, increasing vascular permeability, and ultimately causing plasma extravasation and tissue edema (1) (**Figure 1**).

The genes associated with HAE-nC1-INH include those related to the kallikrein-kinin system, such as *F12* (43), *PLG* (44)*, KNG1* (45)*, HS3ST6* (46), and *CPN1* (11), as well as genes associated with EC function, including *MYOF* (47)*, ANGPT1* (47), and *DAB2IP* (48), have been identified. The identification of these novel pathogenic genes has expanded our understanding of HAE pathogenesis.

The vascular endothelium and circulating cells can initiate, activate, amplify, and regulate both the complement and coagulation systems and respond to the effectors produced by their activation (49). In HAE patients, dynamic changes in the endothelial cell barrier constitute a central mechanism for increased vascular permeability and edema formation. Macromolecules (>3 nm) are primarily transported via caveolae-mediated vesicular transport, whereas smaller molecules cross the endothelial barrier via the paracellular pathway, a process that is dynamically regulated by interendothelial junctions (50).

VE-cadherin is crucial for modulating changes in vascular permeability that are associated with HAE. Its phosphorylation and internalization, regulated by kinases such as Src, can transiently open adherens junctions, increase intercellular gaps, and induce plasma leakage into tissue spaces, ultimately resulting in edema (26). Variations in microvascular architecture and interendothelial junctions across tissues influence the location of HAE-related edema, including the skin, gastrointestinal tract, and airways (51).

Additionally, the resolution of edema relies on lymphatic drainage to clear interstitial fluid, a process that current HAE treatments cannot accelerate. This finding underscores the importance of early intervention in HAE—fluid extravasation must be inhibited before moderate-to-severe swelling develops. Existing HAE treatment strategies largely focus on inhibiting BK release, but the direct regulation of the endothelial barrier function remains underinvestigated.

## 4 Role of ECs in vascular permeability abnormalities and edema mechanisms in HAE

### 4.1 CAS/KKS activation and HAE edema

In HAE, edema results from the dysregulation of the CAS/KKS system, causing excessive BK production or signaling (1).

CAS is a proteolytic system that begins with the autoactivation of factor XII (FXII) on various surfaces, leading to the conversion of PK to PKa. This process involves mutual activation of FXII by PKa, which amplifies their activation and subsequently triggers the activation of factor XI (FXI), initiating the intrinsic blood coagulation pathway. KKS involves the cleavage of high-molecular-weight kininogen (HMWK) by PKa, releasing BK. KKS is involved in a proteolytic cascade that mediates proinflammatory and procoagulant responses (52, 53).

In the contact activation phase of the KKS, FXII undergoes autoactivation upon contact with negatively charged surfaces, triggering a surface-catalyzed zymogen-to-enzyme conversion that ultimately leads to FXII cleavage and the release of its active form, FXIIa (8). Approximately 85% of PKs circulate as a 1:1 complex with HMWK, facilitating their recruitment to the endothelial cell surface (54). Upon activation, PKa further promotes FXII activation, leading to additional FXIIa generation. FXII autoactivation upon contact with activating surfaces is slow, but reciprocal activation between PK and FXII creates a positive feedback loop, accelerating KKS activity (55). Ultimately, the activation cascade results in HMWK proteolysis by PKa, leading to the release of BK, the key kinin in the KKS. KKS activation is tightly regulated by C1-INH, which suppresses FXII autoactivation, inhibits FXII-mediated PK activation, and blocks the positive feedback activation of HMWK and FXII by PKa. Consequently, C1-INH deficiency or dysfunction results in excessive BK production, leading to edema (56).

Although these two systems exist primarily in plasma, some components, such as the PK-HMWK complex, localize to the endothelial cell surface. Its key effector, BK, also acts on endothelial receptors, highlighting the critical role of CAS and KKS in regulating vascular permeability.

HMWK and FXII interact with a multiprotein receptor system on the endothelium in a Zn^2+^-dependent manner, involving the urokinase plasminogen activator receptor (uPAR), globular C1q receptor (gC1qR), and cytokeratin 1 (CK) (57, 58). These three endothelial cell proteins can function independently or form bimolecular complexes with different affinities for HMWK and FXII, thereby modulating KKS activation and altering its interactions with other proteolytic systems (59). Additionally, ECs regulate contact system activation through alternative mechanisms. HMWK binds to glycosaminoglycans on the endothelial surface, inhibiting contact system activation and HMWK cleavage (60, 61). Endothelium-derived products, including heat shock protein-90 (HSP90) and prolylcarboxypeptidase, are thought to positively regulate contact system activation (53, 62, 63) (**Figure 1**).

These events underscore the central role of the endothelium as both a structural barrier and a signaling hub in bradykinin-mediated vascular leakage, establishing it as a key effector in HAE pathogenesis.

### 4.2 BK signaling pathway and endothelial activation

BK channels facilitate vascular leakage by activating G protein-coupled receptors, specifically B2Rs and B1Rs, on endothelial cells through two separate mechanisms. The initial mechanism involves the phosphorylation of VE-cadherin and its linked catenins, resulting in VE-cadherin internalization and degradation, which destabilizes adherens junctions. The second mechanism involves actomyosin contraction via reorganization of the actin cytoskeleton into stress fibers, leading to centripetal contraction of endothelial cells and enlargement of interendothelial pores (64).

**Figure 2:**
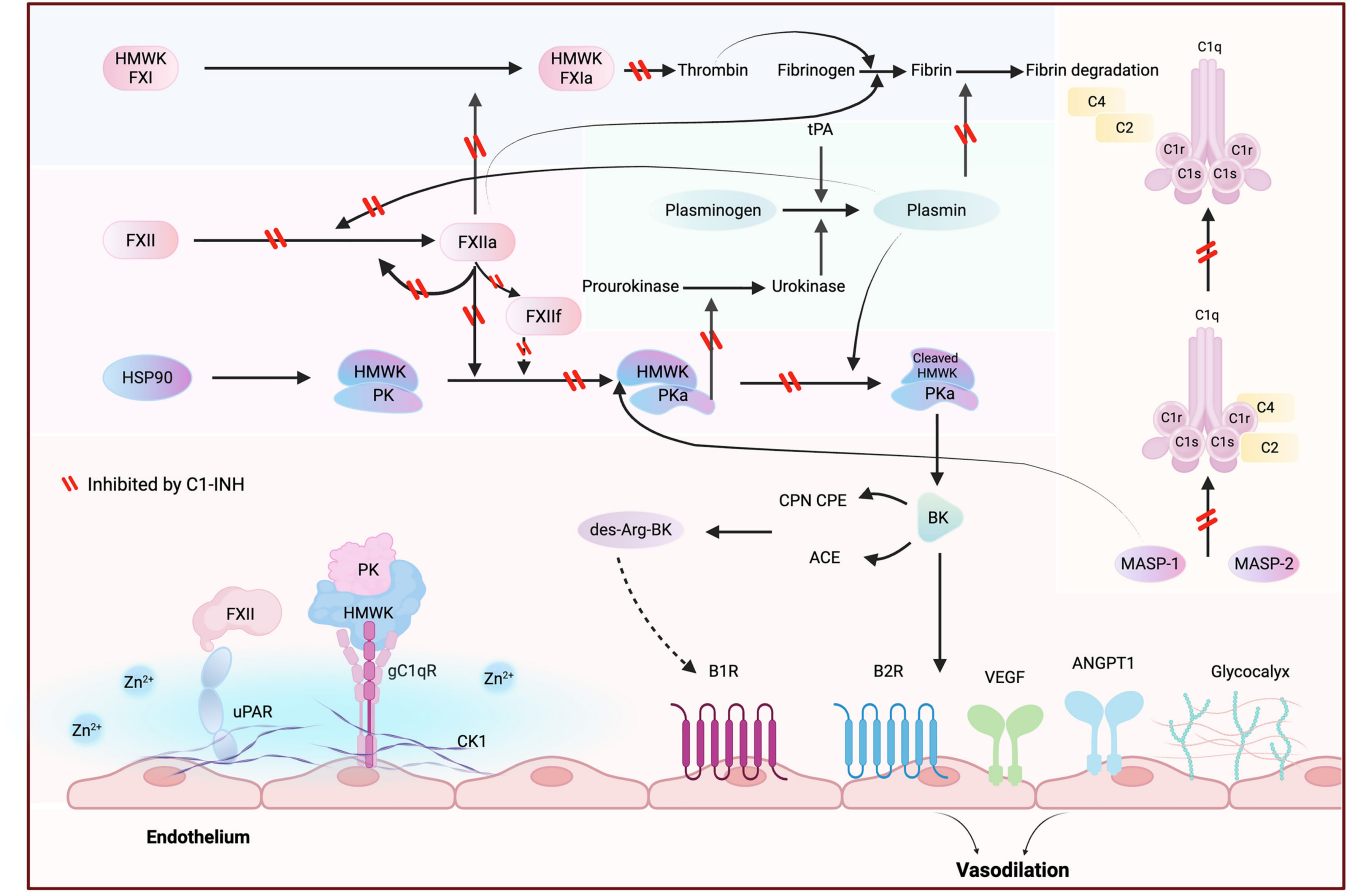
Endothelial signaling pathways regulating vascular permeability and barrier stability relevant to HAE. The schematic summarizes key permeability-promoting pathways triggered by bradykinin and VEGF, and barrier-stabilizing signaling mediated by ANGPT1–Tie2. Bradykinin binding to B2R activates PLC, generating DAG and elevating intracellular Ca²^+^, which together activate PKC. PKC-dependent phosphorylation of junctional components (e.g., occludin/ZO-1 and related tight-junction complexes) contributes to junction destabilization, while Ca²^+^ also activates PLA_2_ to promote PGI_2_ production and stimulates eNOS to generate NO, collectively modulating vascular tone and endothelial permeability. VEGF–VEGFR signaling activates Src and PI3K/Akt and also engages PLCγ-dependent Ca²^+^ signaling, converging on Rac/eNOS and PKC/PLA_2_ pathways to disrupt junctional integrity and promote leakage. In contrast, ANGPT1-activated Tie2 signaling promotes PI3K/Akt-dependent Rac1 activity, supports VE-cadherin–based adherens junction assembly, and suppresses inflammatory activation, thereby strengthening barrier function. The upper panel compares an intact endothelial glycocalyx under laminar flow with glycocalyx degradation under disturbed shear stress, which favors downstream eNOS activation and vasodilation and is associated with increased vascular leakage.

The binding of BK to B2R triggers multiple signaling events, including phospholipase C (PLC) and protein kinase C (PKC) activation, increased intracellular Ca²^+^, and activation of the small GTPase RhoA. B1R signaling similarly activates PLC and PKC while increasing intracellular Ca²^+^ levels (64, 65). Kinin receptor signaling additionally stimulates the release of the vasodilator nitric oxide (NO). Cytosolic endothelial NO synthase (eNOS)-derived NO induces S-nitrosation of junctional proteins, thereby increasing vascular permeability (66). Furthermore, sustained B2R activation upregulates vascular permeability factors such as VEGF, promoting pore formation, endothelial cell activation, and plasma osmotic alterations, collectively exacerbating fluid extravasation through multiple pathways (67, 68) (**Figure 2**).

Although absent in normal tissues, B1R is induced by inflammatory stimuli or B2 receptor-mediated signaling. *In vitro* and *in vivo* studies have demonstrated B1R activation during acute episodes. Unlike rapidly desensitizing B2Rs, B1Rs exhibit slow and incomplete desensitization after agonist binding, potentially prolonging the duration of angioedema. Emerging evidence suggests that B1Rs may play a crucial role in localizing edematous manifestations (69–71). Notably, B2R activation may amplify vascular leakage through proinflammatory effects and B1R upregulation, establishing a synergistic pathogenic cycle that drives characteristic angioedema formation (72, 73).

### 4.3 Endothelial cell activation in HAE

Pathophysiological pathways involved in HAE reveal a plethora of molecules from the vascular endothelium, which may serve as biomarkers. Here, we first discuss the molecular changes in HAE-C1-INH. Endocan and vascular cell adhesion molecule 1 (VCAM-1) are sensitive markers indicating sustained endothelial activation or dysfunction in HAE patients during attack-free periods (74). Soluble VE-cadherin detected in patient serum during an HAE attack could serve as a clinically significant diagnostic biomarker (75).

Elevated endothelial permeability indicates endothelial cell activation. Evaluating the *in vivo* function of ECs is challenging, but the combination of these markers (e.g., von Willebrand factor [vWF], soluble E-selectin [sE-selectin], and endothelin-1 [ET-1]) may accurately reflect the state of EC activation (76).

While previous studies have indicated that the blood levels of ET-1 and vWF are largely normal in HAE patients during interattack periods, increased soluble E-selectin levels are detected during these remission phases (76). E-selectin is exclusively expressed by activated ECs, and its sE-selectin activity primarily originates from enzymatic cleavage or cellular shedding. Certain E-selectin-cleaving enzymes are believed to belong to the serine protease family, while C1-INH, as a potential inhibitor of these enzymes, may help maintain E-selectin stability by suppressing their activity, thereby preventing excessive shedding of adhesion molecules. This mechanism likely involves C1-INH binding to the E-selectin-cleaving enzyme at its cleavage site, inhibiting excessive proteolysis. Additionally, higher sE-selectin concentrations may help protect HAE patients from inflammatory responses (76, 77).

Research has indicated elevated levels of sE-selectin, ET-1, and vWF during HAE attacks (78). vWF is the benchmark for assessing endothelial damage (79). ET-1 is produced by ECs and released basolaterally, but it can also be detected in plasma. It is among the most potent vasoconstrictors and has endothelium-stabilizing effects by inhibiting EC apoptosis (80, 81). Taken together, these findings suggest that ET-1 might contribute to the resolution of HAE attacks. ET-1 and arginine vasopressin act as strong synergistic vasoconstrictors, and their elevation during acute HAE attacks collectively opposes the vasodilatory effects of BK. Moreover, the level of vasodilatory adrenomedullin, which can mitigate BK-induced vascular permeability, is elevated during attacks (73).

Subsequent studies revealed that all known factors driving vasodilation and permeabilization increased during the intercritical phase. Endothelial cell-expressed VEGF-A primarily promotes angiogenesis and increases vascular permeability, whereas VEGF-C contributes mainly to lymphangiogenesis and fluid drainage. They are significantly elevated in patients with more frequent attacks but show no marked difference between the attack and remission phases, suggesting a pathogenetic role as predisposing factors (82, 83). Angiopoietin-2 (Angpt2), a proangiogenic factor produced mainly by ECs, plays a role in regulating endothelial homeostasis. In HAE, Angpt2 levels are elevated, particularly in patients with more frequent attacks (82). Angpt2 interacts with the vascular endothelial tyrosine kinase receptor Tie2 as a negative regulator. While angiopoietin-1 (Angpt1) promotes vascular stability by activating Tie2, Angpt2 inactivates Tie2, increasing endothelial cell sensitivity to VEGF and other inflammatory factors and leading to increased vascular permeability (82, 84). Secreted phospholipase A2 (sPLA2) modulates vascular permeability by directly activating endothelial cells or by catalyzing the synthesis and breakdown of vasoactive molecules (85). One study revealed that sPLA2 is the initial mediator exhibiting contrasting behaviors, increasing during clinical remission and decreasing during angioedema attacks (86).

Nitric oxide (NO) is physiologically released by ECs through endothelial nitric oxide synthase (eNOS). Demirtuk et al. reported that plasma levels of eNOS were elevated in HAE patients during both the remission and attack phases, whereas NO metabolites increased only during attacks. eNOS and its derived NO are crucial in regulating hyperpermeability (87), potentially indicating persistent hyperpermeability in HAE (88). Furthermore, eNOS activities are modulated by several proteins, such as Hsp90, suggesting that the release of Hsp90 following endothelial activation could affect eNOS activity (89). Asymmetric dimethylarginine (ADMA), a circulating peptide, significantly inhibits endothelial NO synthesis, with elevated levels observed in HAE patients (90).

More recent research has also investigated patients with HAE-nC1-INH in remission. Bova et al. reported increased levels of Angpt1, VEGF-A, and VEGF-C in patients with unknown-mutation HAE (U-HAE) and elevated VEGF-C levels in those with FXII-HAE (91). Studies on endothelium-derived vasoactive mediators in HAE suggest that endothelial function is significantly impaired in HAE patients even during asymptomatic periods. Some of these activated molecules warrant further investigation, as they may serve as biomarkers for diagnosing and predicting disease severity in HAE.

Complement activation may contribute to alterations in vascular permeability. Elevated terminal complement complex (TCC) levels are observed during HAE attacks (92). Circulating inactive TCCs have been shown to directly increase endothelial permeability (93).

### 4.4 Glycocalyx damage and permeability regulation

The glycocalyx is a dynamic network that is composed of glycoproteins, proteoglycans, and glycosaminoglycans (GAGs) that covers the luminal surface of ECs, establishing a multifunctional barrier between the vascular wall and circulating blood components. Its established functions include (1) regulating vascular permeability to water and macromolecules (2); mediating mechanotransduction of fluid shear stress and pressure to the endothelial cytoskeleton, as well as modulating shear stress-induced NO production (3); modulating erythrocyte and leukocyte adhesion; and (4) regulating inflammatory responses through the binding of inflammatory cytokines (94) (**Figure 2**).

GAGs, such as heparan sulfate (HS), chondroitin sulfate (CS), and hyaluronic acid, are negatively charged polysaccharides that form proteoglycans when covalently linked to core proteins. Syndecan-1, a transmembrane proteoglycan carrying HS and CS chains, anchors to the cytoskeleton and mediates endothelial mechanotransduction under shear stress (94–96). Notably, HS can bind key components of the contact system, such as FXII, PK, and HMWK, thereby potentially modulating local activation of the CAS/KKS system at the endothelial surface (46, 61).

Several studies have explored the pathophysiological effects of glycocalyx degradation and shedding in conditions such as atherosclerosis, inflammation, sepsis, hypertension, aging, cancer, and diabetes (94, 97–99). Sepsis-induced edema may result from increased vascular permeability caused by endothelial glycocalyx degradation (100). Plaques predominantly develop in atheroprone regions where the glycocalyx is compromised (101). A healthy glycocalyx may positively regulate blood pressure through its barrier function, anti-inflammatory properties, and promotion of NO production, but the specific mechanisms remain unclear. More studies are necessary to determine how the glycocalyx protects against the development of hypertension. Additionally, glycocalyx degradation is associated with preeclampsia (severe hypertension during pregnancy) (102, 103). Research has indicated that endothelial permeability is influenced by specific glycocalyx components. A thinner glycocalyx is typically associated with increased permeability (94).

Previously, the significance of the endothelial glycocalyx was not fully recognized, as it was generally regarded primarily as a protective layer, akin to a sugary coating enveloping cells, and independent of interactions with and regulation of biochemical signaling pathways. With the growing recognition of the crucial role of the glycocalyx in vascular health, endothelial glycocalyx-related molecules are now regarded as potential blood biomarkers (104–106). Nevertheless, research in this field remains limited by the technical challenges associated with glycocalyx imaging and assessment.

Notably, the vascular hyperpermeability that characterizes HAE is closely linked to excess BK generation. *In vitro* studies have shown that BK can reversibly increase glycocalyx permeability without causing overt structural damage in healthy vessels (107). Disruption of specific glycocalyx components may increase endothelial accessibility to plasma kallikrein and coagulation factor XII, thereby facilitating local BK signaling and vascular leakage. In this context, glycocalyx dysfunction is unlikely to serve as the primary trigger of HAE attacks, but rather as a permissive factor that lowers the threshold for edema formation.

Recent evidence further refines this concept by showing that experimental glycocalyx degradation not only weakens endothelial barrier integrity, but also selectively amplifies mediator-induced transendothelial water flux, including that triggered by BK (108). These findings suggest that the glycocalyx acts as an active regulator of edema formation rather than a passive surface coating, and support the idea that glycocalyx injury may shape the magnitude and susceptibility of BK-driven vascular leakage in HAE. Future studies employing intravital imaging may help define the dynamic contribution of glycocalyx alterations during acute attacks.

### 4.5 Conversion of endothelial proinflammatory and procoagulant phenotypes

ECs balance the coagulation process by expressing procoagulant and anticoagulant factors to maintain the fluid state of the blood while rapidly triggering the coagulation cascade in response to vascular injury. ECs have anticoagulant activities, including the release of prostacyclin (PGI_2_) and NO to inhibit platelet activation, the expression of protein C receptors to inhibit coagulation factors Va and VIIIa, and the release of tissue factor pathway inhibitor (TFPI). Additionally, ECs help maintain fibrinolytic system function by releasing tissue plasminogen activator (tPA), thereby preventing abnormal clot formation (14, 109).

Under various pathological conditions, such as infection, inflammation, hypoxia, or trauma, ECs become activated and shift toward a proinflammatory and prothrombotic phenotype. This transition is characterized by reduced secretion of anticoagulant and fibrinolytic factors and increased expression of procoagulant mediators, such as vWF and tissue factor (TF), which facilitate platelet adhesion and trigger the extrinsic coagulation pathway by activating factor X (FX). Moreover, the production of NO, PGI_2_, and tPA decreases, contributing to a hypercoagulable and hypofibrinolytic state (110, 111).

In HAE patients, plasma levels of prothrombin fragment 1 + 2, thrombin-antithrombin complexes, and D-dimer are notably elevated during remission and increase further during attacks. The significantly reduced activated partial thromboplastin time (APTT) suggests abnormal activation of the coagulation pathway, serving as a crucial indicator of the procoagulant state in HAE (112–114). The D-dimer levels are particularly elevated during submucosal attacks, such as those involving the abdomen and oropharyngeal–laryngeal regions, which may reflect organ-specific endothelial activation. Owing to the limited inhibitory activity of C1-INH on FXII and PKa at the endothelial surface, the contact system is more susceptible to localized activation, thereby promoting coagulation (115). More extensive edema across multiple sites is also associated with higher D-dimer levels, further supporting the link between endothelial dysfunction and coagulation system activation.

Notably, despite coagulation system activation, thrombotic events are rarely reported in HAE patients (56, 116, 117). One possible explanation is that during HAE attacks, although FXIIa and the contact system are activated, this process is often accompanied by concurrent activation of the fibrinolytic system, resulting in a dynamic equilibrium between coagulation and fibrinolysis (58). Another proposed mechanism involves ECs, which, upon BK stimulation, may release TF, triggering the extrinsic coagulation pathway. Moreover, they can also secrete tissue-type or urokinase-type plasminogen activator (tPA/uPA), promoting fibrinolytic activity (118).

ECs play a central role in initiating and regulating vascular inflammation. Their shift from a homeostatic to a proinflammatory phenotype is a prerequisite for the recruitment of immune cells across the endothelium. Inflammatory stimuli activate signaling pathways such as NF−κB and MAPK, leading to marked upregulation of the expressions of ICAM−1, VCAM−1, and E−selectin. This remodels the adhesive interface of the vessel wall and drives the classic cascade of leukocyte recruitment: rolling, firm adhesion, and ultimately transendothelial migration (119–121). This adhesion cascade not only determines the spatial and temporal characteristics of immune cell entry into tissues but also serves as a key determinant of focal inflammatory expansion. Simultaneously, endothelial cells, which are stimulated by inflammation, secrete chemokines such as IL-8 and MCP-1, which guide the directional migration of immune effector cells (122). Therefore, the persistence of the proinflammatory phenotype may increase leukocyte infiltration and local endothelial damage. Disruption of endothelial junctions, triggered by this process, can synergize with BK-mediated barrier impairment in HAE to increase vascular permeability. Targeting the proinflammatory endothelial phenotype with nonhormonal therapies is a novel therapeutic approach. In various diseases, improving endothelial function by targeting endothelial oxidative stress and inflammation can significantly increase treatment efficacy (123). ECs in HAE are not merely passive targets but also act as drivers of proinflammatory and prothrombotic phenotype switching.

### 4.6 Vascular endothelial dysfunction-related angioedema

**Figure 3:**
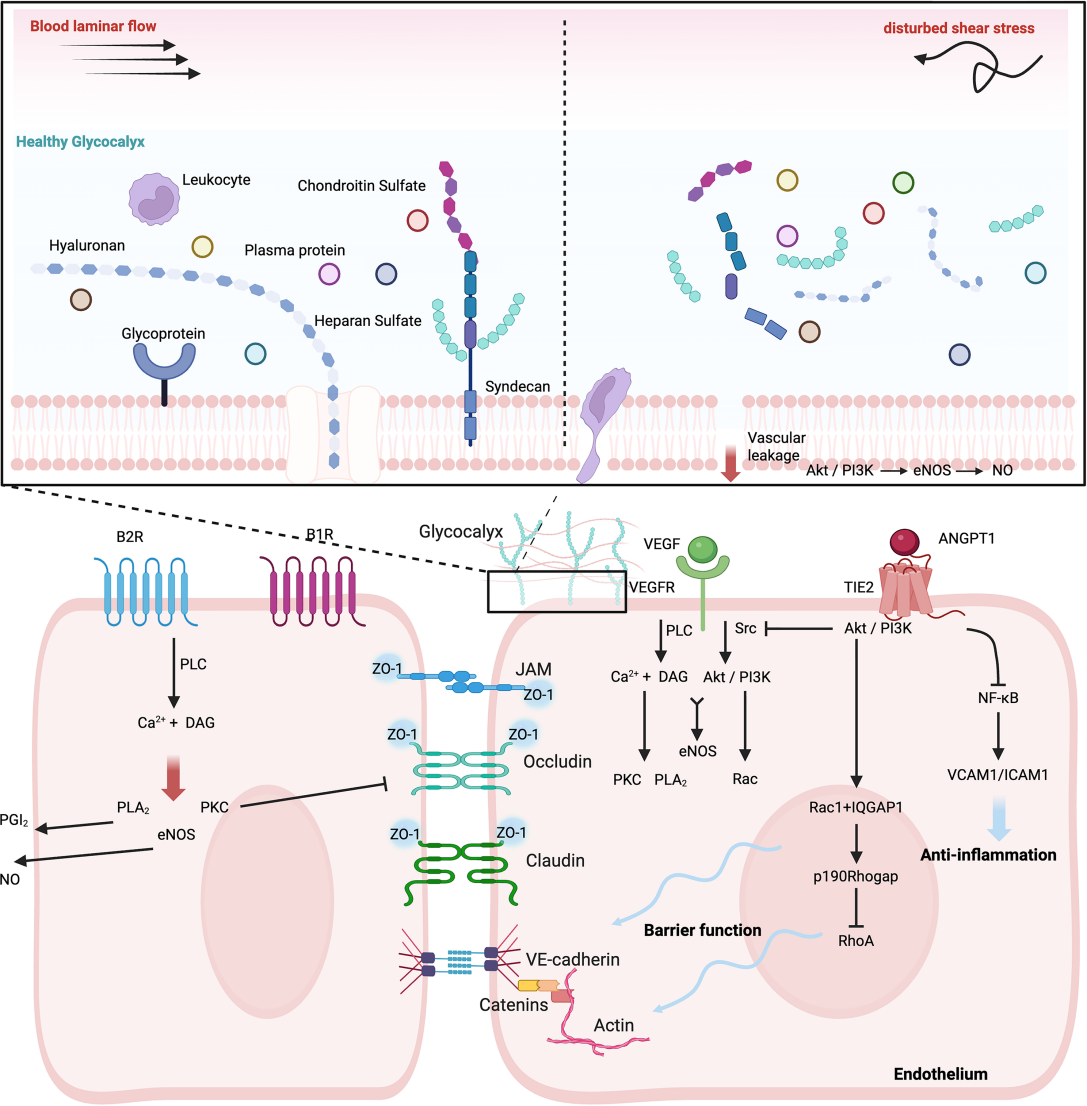
Subtype-specific molecular mechanisms underlying HAE. This figure summarizes subtype-specific molecular mechanisms of HAE that converge on vascular leakage. BK-centered subtypes, including HAE–C1-INH, HAE–FXII, HAE–PLG, and HAE–KNG1, involve dysregulation of the generation phase of the kallikrein–kinin system, increasing bradykinin availability and B2R signaling to enhance vascular permeability. HAE–KNG1 affects this generation phase by altered bradykinin release and processing from kininogen. In contrast, HAE–CPN is associated with reduced bradykinin inactivation, prolonging bradykinin activity. Beyond the kinin pathway, HAE–ANGPT1 is characterized by impaired ANGPT1–Tie2 signaling that weakens vascular stabilization, whereas HAE–MYOF and HAE–DAB2IP are associated with enhanced VEGF–VEGFR2 signaling that promotes permeability. HAE–HS3ST6 involves heparan sulfate–related glycosaminoglycan modification and kininogen–surface interactions. C1-INH, C1 inhibitor; HMWK, high molecular weight kininogen; FXII, coagulation factor XII; PK, prekallikrein; PKa, activated plasma kallikrein; tPA, tissue-type plasminogen activator; HSP90, heat shock protein 90; MASP, mannose-binding lectin–associated serine protease; BK, bradykinin; des-Arg-BK, des-arginine bradykinin; CPN, carboxypeptidase N; CPE, carboxypeptidase E; ACE, angiotensin-converting enzyme; uPAR, urokinase plasminogen activator receptor; CK1, cytokeratin 1; gC1qR, receptor for the globular heads of C1q; B2R, bradykinin receptor type 2; B1R, bradykinin receptor type 1; VEGF, vascular endothelial growth factor; VEGFR, vascular endothelial growth factor receptor; ANGPT1, angiopoietin 1; PLC, phospholipase C; DAG, diacylglycerol; PLA_2_, phospholipase A_2_; eNOS, endothelial nitric oxide synthase; NO, nitric oxide; PKC, protein kinase C; PGI_2_, prostacyclin; Akt/AKT, protein kinase B; PI3K, phosphoinositide 3-kinase; JAMs, junctional adhesion molecules; VE-cadherin, vascular endothelial cadherin; ZO-1, zonula occludens-1; HAE–C1-INH, HAE due to C1-INH deficiency; HAE–FXII, HAE due to factor XII mutation; HAE–PLG, HAE due to plasminogen mutation; HAE–KNG1, HAE due to kininogen 1 mutation; HAE–CPN, HAE due to carboxypeptidase N deficiency; HAE–ANGPT1, HAE due to angiopoietin-1 mutation; HAE–MYOF, HAE due to myoferlin mutation; HAE–DAB2IP, HAE due to disabled homolog 2 interacting protein mutation; HAE–HS3ST6, HAE due to heparan sulfate 3-O-sulfotransferase 6 mutation.

In the DANCE study by Reshef et al. (13), 91 experts from 35 countries developed a framework for defining, naming, and classifying angioedema into five main subtypes: mast cell-mediated (AE-MC), BK-mediated (AE-BK), vascular endothelium dysfunction-related (AE-VE), drug-induced (AE-DI), and unknown etiology (AE-UNK). With the recent discovery of novel HAE subtypes, including HAE-DAB2IP (48) and HAE-CPN1 (11), an endotype-based classification paradigm has been proposed: BK-mediated HAE and VEGF-mediated HAE (124). Under the current categorization (13), HAE-C1-INH, HAE-FXII, HAE-PLG, and HAE-KNG1 are classified as AE-BK, whereas HAE-ANGPT1, HAE-MYOF, and HAE-HS3ST6 are classified as AE-VE. Mechanistic studies on the genetic basis of HAE-nC1-INH, remain limited. Given that these variants have so far been reported mainly in single families or limited pedigrees, they should be interpreted as rare but mechanistically informative findings that expand the endothelial-related spectrum of HAE. These findings support endothelial dysfunction as an important entry point for investigating HAE subtype classification and pathogenic mechanisms (**Figure 3**).

#### 4.6.1 HAE-ANGPT1

In 2018, a c.355G>T (p.Ala119Ser) missense variant in the *ANGPT1* gene was identified in affected female members of an Italian family. This mutation disrupts the interaction between ANGPT1 and its membrane receptor TIE2 (125). The ANGPT1-TIE2 pathway is crucial for endothelial stability and barrier function regulation (126, 127). ANGPT1 regulates endothelial barrier integrity by inhibiting multiple permeability-enhancing factors, including VEGF (128). ANGPT1 preserves adult vasculature integrity and prevents plasma leakage (129).

ANGPT1 is a ligand of the TIE2 receptor and is predominantly expressed in developing vascular ECs (130). ANGPT1 features a C-terminal fibrinogen-like domain for receptor binding, a central coiled-coil domain that oligomerizes fibrinogen-like domains, and a short N-terminal domain that aggregates oligomers into multimers of varying sizes (130, 131). Unlike most known growth factors, ANGPT1 requires a multimeric structure to effectively activate TIE2, whereas dimers and monomers exhibit significantly lower binding capacities (131, 132).

In HAE patients, compared with proteins from unaffected relatives, mutant *ANGPT1* has a reduced multimerization capacity and weaker binding affinity for the TIE-Fc chimera. Experimental findings suggest that the p.A119S variant results in greater functional impairment (125). Overall, dysfunction of the ANGPT1-TIE2 axis in ECs may represent a novel pathophysiological mechanism of U-HAE, highlighting TIE2-targeted therapies as a potential strategy to restore endothelial barrier function and reduce vascular leakage.

#### 4.6.2 HAE-MYOF

In 2020, a rare missense mutation in the *MYOF* gene (c.651G>T, p.Arg217Ser), designated a gain-of-function variant (MYOF-217S), was initially identified in an Italian family (47). The MYOF gene encodes myoferlin, which is highly enriched at the plasma membrane of ECs and regulates VEGF signaling by modulating VEGF receptor-2 (VEGFR2) levels. VEGFR2 is the principal tyrosine kinase receptor that mediates most VEGF functions in ECs (133).

Research has indicated that myoferlin interacts with dynamin-2 and VEGFR2 to inhibit CBL-mediated polyubiquitination and proteasomal degradation of VEGFR2, thereby stabilizing VEGFR2 expression (47, 134). Functional validation through *in vitro* studies demonstrated that mutant myoferlin augments VEGFR2 membrane localization upon VEGF stimulation, leading to hyperactivation of VEGFR2 signaling pathways and contributing to the onset of angioedema (47).

#### 4.6.3 HAE-HS3ST6

Bork et al. reported the *HS3ST6* mutation c.430A>T (p.Thr144Ser) in a family with HAE and normal C1-INH, which led to incomplete synthesis of HS (46). Among the proteoglycan core proteins linked to HS, the syndecan family is included, with syndecan-2 being the most abundant in ECs. The overexpression of syndecan-2 significantly increased the total cell-surface HS content, thereby increasing the numbers of HMWK binding sites (135). HMWK can also attach to the endothelial cell surface through interactions with multiple binding partners, including gC1qR and cytokeratin 1 (59, 136, 137). This specific binding protects HMWK from PKa-mediated cleavage, thereby limiting BK generation. In addition, HS proteoglycans mediate the endocytosis of HMWK, regulating both the generation of BK and its distribution on the cell surface (138–140).

However, the aforementioned mutation results in defective O-sulfation of HS on syndecan-2, impairing HMWK binding and endocytosis at the endothelial surface. Consequently, HMWK tends to remain on the membrane and interact with alternative partners, increasing its susceptibility to cleavage by PKa. This leads to excessive BK production and ultimately triggers the onset of angioedema (46).

### 4.6.4 HAE-DAB2IP

A novel missense variant (p.D239N) in the *DAB2IP* gene was identified in an Argentine family. This mutation occurs within the C2 domain, affecting the interaction between DAB2IP (DAB2 interactive protein) and VEGFR2, leading to a loss of protein function and disruption of endothelial VEGF/VEGFR2 signaling (48).

DAB2IP is highly expressed in ECs and regulates cellular responses to extracellular signals. Research has indicated that DAB2IP acts as an intrinsic inhibitor of pathological angiogenesis by inhibiting VEGFR2 activity and its downstream signaling pathways (141). The VEGF-A/VEGFR2 pathway plays a pivotal role in angiogenesis, regulating vascular permeability and promoting inflammatory responses (142, 143). During the late phase of VEGF-induced signaling, DAB2IP is recruited to the VEGFR2-PI3K complex, where it binds to the cytoplasmic domain of VEGFR2 via its C2 domain, counteracting VEGF-driven signal transduction (144).

*In vitro* studies confirmed that the p.D239N substitution leads to loss of DAB2IP function, affecting DAB2IP protein stability and its ability to bind VEGFR2, thereby impairing VEGFR2 inactivation and downstream signaling suppression. Given that VEGF/VEGFR2 hyperactivation promotes inflammation through increased vascular permeability, these findings further support that dysregulated VEGF-mediated signaling disrupts endothelial permeability control, contributing to the recurrence of angioedema.

On the basis of this evidence, reduced interaction between DAB2IP and VEGFR2 may lead to loss of PI3K–AKT axis inhibition, which in turn impairs endothelial barrier function and contributes to the pathogenesis of vascular leakage and angioedema (48).

## 5 Therapeutic strategies targeting endothelial cell dysfunction

The past decade has witnessed significant advancements in therapeutic options for HAE, with the approval of targeted therapies for both acute attacks and long-term prevention, enabling more precise and individualized management. As therapeutic options continue to expand, adjusting treatment strategies on the basis of individual responses has become an important trend in clinical practice.

The primary mechanisms of existing marketed drugs include C1-INH replacement, kallikrein inhibition, BK receptor antagonism, and androgen use. Approved long-term prophylactic drugs include plasma-derived C1-INH, the monoclonal antibody lanadelumab, which targets PKa, and the orally administered small-molecule PKa inhibitor berotralstat. Late-phase investigational agents include garadacimab (a factor XIIa-targeting monoclonal antibody) and donidalorsen (a PK synthesis-inhibiting antisense oligonucleotide). The B2R antagonist icatibant can reverse vascular leakage during acute attacks of HAE (145–148). Although the mechanisms through which BK influences endothelial cells are well studied, treatment strategies for HAE that focus on endothelial protection remain underexplored. While most currently approved therapies target upstream components of the kallikrein–kinin system, they do not directly restore or reinforce the integrity of the vascular endothelial barrier. Furthermore, in a subset of patients with HAE-nC1-INH, the underlying pathophysiological mechanisms remain poorly understood, resulting in the limited therapeutic efficacy of existing treatments. Consequently, increasing attention has been given to therapeutic strategies that specifically target endothelial stability and the regulation of vascular permeability. **Table 1** summarizes the therapeutic roadmap from BK-targeted therapy to endothelial barrier stabilization in HAE.

**Table 1:** Therapeutic roadmap from BK-targeted therapy to endothelial barrier stabilization in HAE.

Therapeutic strategy/agent	Stage	Main mechanism	Most relevant HAE context	Key unknowns
C1-INH replacement	Approved	Restores C1-INH activity; reduces CAS/KKS activation and BK generation	HAE-C1-INH	Direct endothelial barrier effects remain unclear
Contact system/BK pathway blockade, including lanadelumab, berotralstat, icatibant, garadacimab, and donidalorsen	Approved	Reduces BK generation or blocks BK-induced B2R signaling	Mainly BK-mediated HAE, especially HAE-C1-INH	Residual endothelial vulnerability despite BK control
CU06-1004	Preclinical in HAE	Stabilizes endothelial junctions and prevents BK-induced interendothelial gap formation	HAE with endothelial barrier disruption	Clinical efficacy, safety, and responsive phenotypes
HMWK/gC1qR-targeted approaches	Preclinical/conceptual	Modulates endothelial surface assembly of BK-generating complexes	BK-mediated angioedema; selected HAE-nC1-INH mechanisms	Specificity, feasibility, and *in vivo* validation
Glycocalyx protection/restoration, represented by sulodexide	Conceptual for HAE	Preserves/restores endothelial glycocalyx and microvascular barrier function	HAE with endothelial or glycocalyx vulnerability	Direct HAE evidence and biomarkers for patient selection

CU06-1004, a cholesterol-derived pseudosugar, acts as an endothelial dysfunction blocker by safeguarding the endothelium against various permeability mediators, such as VEGF, histamine, and IL-1β, through the cAMP/Rac/cortactin pathway (149). This compound inhibits vascular permeability by preventing ligand-induced stress fiber formation while strengthening adherens junctions and tight junctions. Preclinical models have confirmed its efficacy in diseases associated with vascular leakage, including cerebral ischemia, myocardial infarction, cancer, and inflammatory bowel disease (150). Research has demonstrated that CU06–1004 mitigates vascular hyperpermeability in HAE murine models by preventing interendothelial gap formation. This is achieved by inhibiting BK-induced disruption of endothelial cell junction integrity and changes in contractile forces. Importantly, CU06–1004 enhances endothelial integrity without targeting specific stimuli, indicating its potential therapeutic use for various HAE subtypes (151).

In preclinical models, gC1qR is crucial for the biochemical pathway that leads to BK production, resulting in vascular leakage and inflammation (152). These findings underscore the significance of HMWK binding sites and gC1qR as key interaction points in therapeutic strategies for angioedema. The endothelial glycocalyx, a key regulator of microcirculatory perfusion, is expected to become a new biomarker for evaluation and a therapeutic target. Sulodexide, a naturally purified glycosaminoglycan, can restore the endothelial glycocalyx layer. Furthermore, its therapeutic application in COVID-19 patients has been confirmed to effectively replenish the glycocalyx, restore endothelial function, and prevent thrombus formation (153).

## 6 Discussion

In recent years, increasing evidence has highlighted the central role of ECs in the development of edema in HAE. The traditional view holds that HAE is driven primarily by BK-mediated vascular hyperpermeability. However, current evidence indicates that ECs are not only targets of BK signaling but also key regulators of vascular barrier homeostasis, inflammation, and coagulation balance.

BK activates B1/B2 receptors, leading to the phosphorylation, internalization, and degradation of junctional proteins such as VE-cadherin, resulting in cytoskeletal rearrangement and endothelial barrier failure. Beyond intercellular junctions, the endothelial glycocalyx constitutes an additional regulatory layer of vascular permeability. When degraded, it not only weakens barrier integrity but may also amplify BK-driven leakage, supporting the concept that alterations of the endothelial surface layer can shape edema susceptibility rather than merely reflect downstream damage. Moreover, some HAE subtypes harbor pathogenic variants that directly disrupt endothelial signaling pathways such as ANGPT1-TIE2 and VEGFR2, suggesting that endothelial dysfunction itself may serve as an independent pathogenic mechanism in selected subtypes. Together, these findings support an endothelial susceptibility framework of HAE, in which edema-promoting mediators such as BK act on a vascular barrier whose baseline competence may influence the threshold, localization, severity, and recurrence of attacks.

The localized nature of HAE attacks remains controversial. Local BK production (e.g., increased BK levels in the venous system of the affected limb) supports the local trigger hypothesis (154), whereas elevated cleaved HK levels during multisite attacks suggest systemic activation (69). The heterogeneity of ECs may be a key factor. ECs in different vascular beds may exist in a pre-activated state driven by factors such as Angpt2 and VEGF, together with upregulation of B1Rs, which may help explain edema localization (155). Additionally, in the context of C1-INH deficiency, ECs are chronically exposed to a high-permeability environment, and the combination of occasional triggering factors exceeding a threshold may explain the unpredictable nature of edema attacks.

Although current treatments effectively inhibit BK production, they do not directly restore endothelial barrier function. Therapeutic strategies aimed at re-establishing endothelial homeostasis, including glycocalyx preservation or repair and maintenance of intercellular junction integrity, may therefore represent a promising complementary therapeutic strategy. In parallel, combined assessment of endothelial biomarkers may improve the evaluation of disease activity, endothelial injury, and therapeutic response.

Future research should further explore endothelial responses across tissue microenvironments, interactions between endothelial barrier-related factors and the BK pathway, and the dynamic balance between procoagulant and anticoagulant endothelial phenotypes. Establishing endothelial functional biomarker panels reflecting glycocalyx integrity, coagulation–fibrinolysis balance, inflammation regulation, and vascular protection will aid patient stratification, risk prediction, and development of endothelium-targeted interventions. More broadly, the endothelial surface may serve as a convergence point for complement, CAS/KKS, and coagulation pathways (116), providing insights into HAE and related disorders characterized by endothelial hyperpermeability.

Several limitations should be acknowledged. As a narrative review, this article was based on a targeted rather than systematic literature search, with a focus on studies related to HAE, BK-mediated vascular leakage, endothelial dysfunction, glycocalyx disturbance, vascular permeability regulation, and endothelium-associated HAE subtypes. Therefore, no formal meta-analysis or risk-of-bias assessment was performed.
